# Real-time quality feedback on Doppler data for community midwives using edge-AI

**DOI:** 10.1088/3049-477X/ae1bad

**Published:** 2025-11-18

**Authors:** Mohsen Motie-Shirazi, Sepideh Nikookar, Mohammad Ahmad, Alireza Rafiei, Reza Sameni, Peter Rohloff, Gari D Clifford, Nasim Katebi

**Affiliations:** 1 Department of Biomedical Informatics, Emory University, Atlanta, GA, United States of America; 2 Department of Biomedical Engineering, Georgia Institute of Technology, Atlanta, GA, United States of America; 3 Center for Indigenous Health Research, Wuqu’ Kawoq—Maya Health Alliance, Tecpán, Guatemala

**Keywords:** mHealth, Doppler ultrasound, signal quality, machine learning, edge computing, fetal health monitoring, low-resource settings

## Abstract

This study presents a technical framework for real-time fetal Doppler data quality assessment using deep learning and edge-AI, designed to improve data collection and support future clinical studies in low-resource settings. Integrated into a low-cost, edge-computing system co-designed with Indigenous midwives in rural Guatemala, our solution utilizes an Android phone for data acquisition and decision support. Retrospective analysis demonstrates the potential to detect fetal growth restriction, hypertension, and other pregnancy-related conditions using Doppler-based fetal cardiac signals. To ensure accurate assessments and provide immediate feedback, a real-time signal quality metric is essential. We analyzed two fetal Doppler datasets: 191 recordings, captured in rural Guatemala, for training and validation, and five captured in a German hospital (in Leipzig) for testing. The data were segmented into 3.75 s intervals, and categorized into five quality levels: good, poor, radiofrequency interference, talking, and silent. A deep neural network was trained on these segments, achieving a micro $F1$ score of 97.4% and a macro $F1$ score of 94.2%, with 99.2% accuracy for ‘Good’ quality in the Guatemala dataset, based on five-fold cross-validation. For the Leipzig dataset, the $F1$ score was 93.3% on ‘Good’ quality segments, demonstrating the model’s ability to generalize across different datasets. By implementing the algorithm within an Android decision-support application in an mHealth framework, we have enabled real-time feedback during signal acquisition, improving data quality at the source. This scalable, edge mHealth solution offers significant potential to enhance maternal and fetal health monitoring in the Global South, contributing to global health efforts through the integration of mobile technology, AI, and healthcare.

## 1. Introduction

While medical advancements have significantly decreased mortality rates in many countries, childbirth remains perilous for both mother and child. Maternal mortality is as high as 1000 per 100 000 live births in some countries and regions [1]. Annually, there are nearly 2 million stillbirths and 2.3 million children die in the first month of life [2, 3], predominantly in low and middle-income countries (LMICs) and the Global South. Notably, LMICs constitute around 98% of these perinatal deaths, which can be attributed to factors such as the dearth of trained medical professionals, fractured communication pathways and inconsistent data recording, high medical equipment costs, and the subsequent limited access to specialized prenatal care, especially in remote areas [4–7]. In many regions, this situation is not improving, and for some communities, particularly minorities, it is becoming worse [8, 9].

Fetal growth restriction (FGR) and congenital abnormalities are significant contributors to these mortality rates [10, 11]. Proactive identification and prompt intervention can greatly reduce the risks of neonatal morbidity and mortality [12]. In high-income countries, ultrasound imaging, especially fetal ultrasonography for diagnosis of FGR, is the standard method for fetal health monitoring. However, high device costs (from $2000 to $60 000) and the requirement for specialized training, hinder their widespread adoption in LMICs [13]. There is a critical need for scalable, low-cost solutions that can operate effectively in remote areas with spotty telecommunication connectivity, to advance maternal and fetal health in underserved regions.

### 1.1. mHealth system for perinatal monitoring

Over the past decade, a cost-effective mobile health (mHealth) system has been developed and implemented, leveraging low-cost smartphones to collect key data during routine wellness visits [14, 15]. The system was co-designed with traditional Indigenous midwives in rural Guatemala who serve a Maya population with a history of racial/ethnic discrimination and a consequent lack of trust in medical (and other) authorities [16]. Central to this system is a low-cost ($10) one-dimensional Doppler ultrasound (1D-DUS) transducer, which facilitates the monitoring of fetal cardiac activity, a vital measure for identifying potential fetal abnormalities [17]. This transducer connects to smartphones through a standard audio cable, creating a smartphone-based solution that supports effective pregnancy monitoring in remote areas by enabling on-device data collection and analysis [18]. Unlike cardiotocography (CTG), which provides only an averaged heart rate trace derived from ultrasound autocorrelation, raw Doppler preserves richer spatial and temporal information on cardiac mechanics and blood flow at high resolution, making it particularly suitable for machine learning and deep learning (DL) methods that can exploit these complex patterns.

**Figure 1.**
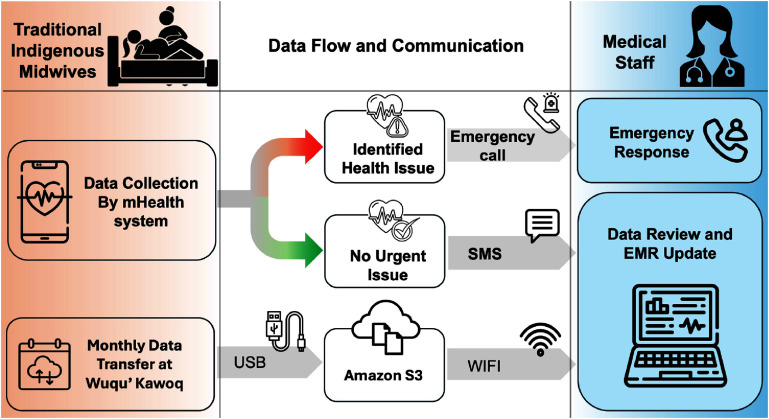
Overview of the current mHealth system workflow designed for traditional Indigenous midwives in rural Guatemala. The system facilitates data collection, health assessment, and communication with medical staff to ensure timely monitoring and support for maternal and fetal health [14, 19].

As illustrated in figure 1, the mHealth system incorporates a structured data collection and transfer workflow. This workflow enables midwives to gather Doppler data and promptly communicate with medical staff, initiating an emergency call if an urgent health issue is detected. For routine cases, a summary is sent by SMS for remote monitoring. Monthly, the data is securely backed up to cloud storage at Wuqu’ Kawoq using USB and Wi-Fi transfer, ensuring accessibility for further review by medical staff, who can update electronic medical records and provide necessary follow-up. This workflow strengthens communication between midwives and healthcare providers, ensuring timely interventions and comprehensive monitoring.

Previous studies have demonstrated that 1D-DUS signals can reliably estimate fetal heart rate, enabling detection of tachycardia and bradycardia, and with further validation may contribute to assessments of gestational age or growth abnormalities [20–22]. These findings highlight the potential of Doppler-based approaches to support earlier identification of fetal cardiac abnormalities or FGR, thereby facilitating timely referral for clinical evaluation and management. However, such applications remain at the research stage and require validation in larger and more diverse cohorts before clinical adoption. Importantly, independent of these exploratory applications, the broader mHealth system in which this technology is embedded has already contributed to significant reductions in maternal mortality within the Indigenous population [19]. Although recordings of up to 20 min were initially used in early field studies [19], later analyses showed that reliable information can be obtained from much shorter segments of approximately 2–5 min [21], which are feasible in routine midwife visits. Furthermore, studies such as Hoyer *et al* [23] demonstrated that identification of fetal state at the time of recording improves the accuracy of downstream analyses, even when the fetus only exhibits one state during recording. This suggests that extended recordings to capture both active and quiet sleep states within a single session are not always necessary.

Despite these advances, the analysis of the Doppler data often happens days, or even weeks after the midwife has captured it on the mHealth app, because connectivity is spotty and weak, so that large files (like the Doppler data) cannot immediately be transmitted for analysis. As shown in figure 1, data backups happen monthly via USB, adding further delays before the information can be reviewed by medical staff. Consequently, low-quality data may only be recognized long after the patient visit, necessitating a repeat recording, and any potential clinical concerns are therefore not addressed in a timely manner. The work in this paper addresses these delays and data quality issues by moving the data to the edge so that low quality data can be flagged in real time, and any critical issues can be identified immediately.

### 1.2. Challenges in 1-D Doppler signal quality

The quality of the input signal is essential in ensuring accurate assessments of physiology. Historically, the quality of the recorded signals, even after provider training, has posed challenges. Notably, approximately 40% of the recordings made by participating midwives in a prior study were of low quality [15]. The diminished signal quality can be attributed to diverse types of noise and interference. Common issues include incorrectly inserting the audio cable connection so that the external microphone did not engage, and the resultant audio was either silent or contained ambient sounds from sources like human conversations and animal noises. Moreover, interference from electronic devices, particularly mobile phones, presents another significant challenge. Most of these issues cannot be easily addressed by post-processing solutions, highlighting the critical need for a system that delivers error messages, real-time feedback and recommendations during the data collection process.

Efforts to classify the quality of DUS signals and identify data that should be ignored have been ongoing. Notably, high-quality DUS signals often exhibit a consistent relative periodicity of fetal heartbeats. This characteristic has prompted past research to suggest DUS quality evaluations based on the repeated presence of these signal patterns. In these works, essential signal features are typically extracted and fed into machine learning algorithms for signal classification.

In one of the earliest studies, a DUS signal quality index (SQI) was introduced, utilizing sample entropy and the relative wavelet energy distribution within a logistic regression model framework [24, 25]. This approach enabled differentiation between high- and low-quality signals (as determined by expert consensus), achieving an impressive accuracy of 95%. In a subsequent study, four SQIs were extracted for each fetal heartbeat segment from 1D-DUS signals collected from 17 subjects [26]. These SQIs were then integrated with sample entropy and a power spectrum density ratio, achieving an accuracy of 85%for classifying expert-annotated data in a five-class task. More recently, a two-step classifier that combined logistic regression and a multiclass support vector machine (SVM) was utilized to analyze 195 fetal recordings [27]. Recordings were split into 0.75 s segments and classified by three annotators into five quality categories. From five such segments, 3.75 s windows were created. For each, 88 features were extracted and later refined to 17 via feature selection. Micro-averaged and macro-averaged $F1$ scores of 96.0% and 94.5%, respectively, were reported. In a more recent study, unsupervised learning techniques like variational autoencoders and self-organizing maps were employed to generate a combined SQI [28]. This method focused on refining fetal heart rate estimates by adjusting for low-quality DUS signals and improving robustness. Since it did not rely on any labeled data for signal quality, no accuracy metrics for signal quality classification were provided. However, integrating signal quality information significantly reduced errors in fetal heart rate estimation.

### 1.3. Study objective

While prior studies have shown great promise in DUS signal quality classification, the use of handcrafted features and conventional machine learning techniques can lead to complex code with slow run times. Modern machine learning provides the potential to further push the boundaries of accuracy and generalization, while reducing forward execution time. The process of manually identifying optimal features for machine learning can sometimes limit the detection of complex signal patterns intrinsic to the data. In contrast, DL models, such as convolutional neural networks (CNNs) and recurrent neural networks (RNNs), have demonstrated the capability to autonomously learn directly from raw data across different applications. Considering the sequential and dynamic nature of DUS signals, DL methods may excel at discerning subtle features and patterns. This potential has been highlighted in prior research on estimating gestational age from DUS, where deep neural networks demonstrated impressive performance improvements with an average error of less than 0.8 months from data with a 1-month resolution [21]. Moreover, as the volume of DUS data continues to grow, the scalability of DL models offers a distinct advantage. Another notable advantage of DL frameworks, such as TensorFlow Lite (TFLite), is their easy adaptability to edge computing systems such as the mobile phone.

**Figure 2.**
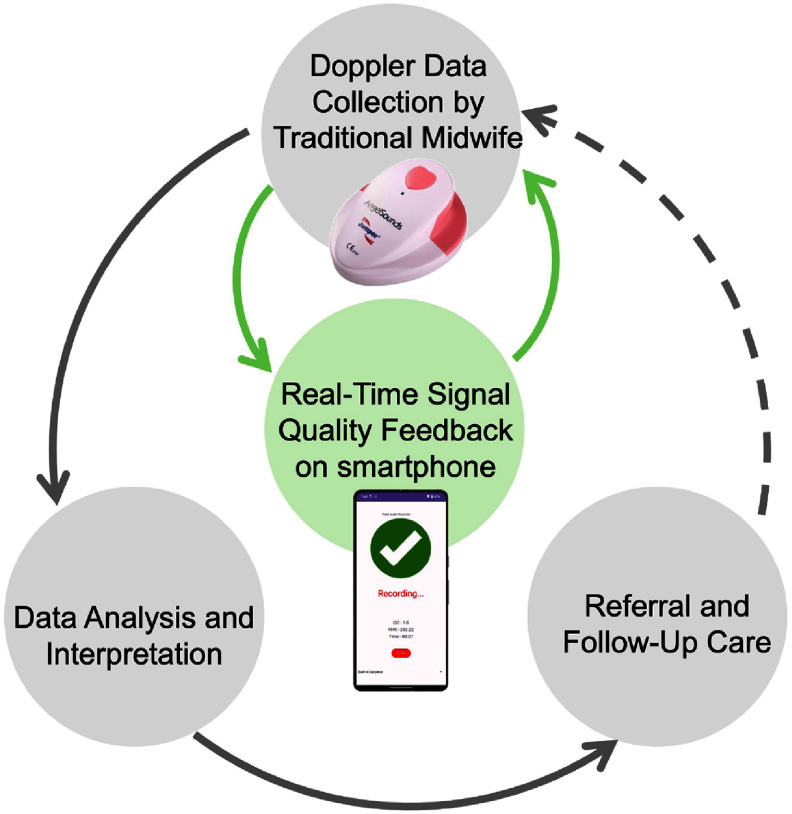
mHealth system for fetal monitoring in low-resource settings. Traditional midwives use a Doppler device with real-time feedback on a smartphone, ensuring higher-quality data capture for accurate analysis, referrals, and follow-up care.

We therefore aimed to develop a DL for 1D-DUS signal quality assessment classifier that can continue to learn from new data, and can run in real-time on a smartphone. As illustrated in figure 2, this mHealth system supports midwives in low-resource settings by providing immediate feedback on signal quality during Doppler recording, enabling them to make real-time adjustments that improve data accuracy and enhance the overall quality of maternal and fetal health monitoring. This real-time feedback not only is likely to lead to much improved data quality but can also act as an instantaneous educational tool for providers, guiding them on the improved positioning of the 1D-Doppler device to obtain higher quality signals. Thus, we expect to increase the information extracted from 1D-DUS devices and enhance their utility, ultimately contributing to better monitoring and improved outcomes in low-resource settings. It is important to note that this study focuses on demonstrating the technical feasibility of real-time Doppler signal quality assessment rather than direct clinical outcomes, which will require separate prospective validation.

## 2. Methods

### 2.1. Data collection

We used two datasets in this study, as summarized in table 1.

**Table 1.** Summary of datasets used for training and testing the 1D-DUS signal quality classification model.

Dataset	Number of subjects	Gestational age range (Weeks)	Classes	Number of 3.75 s windows in each class					Use
Guatemala	142	20–40	Good, poor, interference, talking, silent	22 008	9874	1626	5974	17 260	Training

Leipzig	5	20–27	Good, poor, interference, talking, silent	721	621	0	0	317	Testing

#### 2.1.1. Guatemala dataset

This data originated from a randomized controlled trial in Tecpán, Chimaltenango, a rural highland region of Guatemala. The trial was approved by the Institutional Review Boards of Emory University, Wuqu’ Kawoq $\|$ Maya Health Alliance, and Agnes Scott College (Reference numbers IRB00076231, 2015 001 and 02.02.2015 respectively) and registered on ClinicalTrials.gov (identifier NCT02348840). More details on the design and implementation of the data collection system, and the training of the traditional Indigenous midwives can be found in [14, 15], and [19].

During the trial, 19 midwives utilized a handheld 1D-DUS device (AngelSounds Fetal Doppler JPD-100 s, Jumper Medical Co., Ltd. Shenzhen, China) with a transmission frequency of $3.3\,\mathrm{MHz}$. The device was connected to a smartphone via a bespoke audio cable modified with a capacitor-resistor network to simulate the action of plugging in a hands-free kit to the device (which caused Android to use the external input as a microphone).Data were saved as 16-bit uncompressed WAV files with a 44.1 kHz digitization sampling frequency. Additionally, the DUS device was connected to a speaker, which enabled real-time audio checks by the midwives and helped to verify the DUS was focused on the fetus when the fetal heartbeats were audible. A bespoke app on the low-cost smartphones guided the midwives through a range of tasks. This application not only compiled crucial medical data and captured ultrasound recordings but also integrated an alert system for serious concerns, prompting immediate contact with healthcare personnel. The app also streamlined data communication and archiving via phone calls, SMS, GPRS, and WiFi. To effectively use this setup, midwives participated in four half-day training sessions, followed by an exam to assess proficiency.

The Guatemala data comprises 191 1D-DUS signals recorded from 142 singleton pregnancies, all of Indigenous Maya women in their second to third trimesters. The median duration of the recordings was found to be 10.2 min, with an interquartile range of 1.3 min. More detailed information on the recordings can be found in [27].

#### 2.1.2. Leipzig dataset

This dataset was collected at Leipzig University Hospital (LUH) in Germany. It encompassed data from five volunteers experiencing pregnancies ranging from the 20th to the 27th week of gestation, encompassing diverse scenarios like FGR, premature rupture of membranes, or fetal heart failures. Approval for the study was granted by the LUH ethics committee (record 348-12-24 092 012), and explicit written consent was obtained from each participant. Clinicians concurrently recorded indirect abdominal fetal ECG, a maternal ECG reference, and a 1D-DUS signal for each subject.

### 2.2. Annotation of 1D-DUS recordings

#### 2.2.1. Guatemala dataset

Each 1D-DUS recording was decimated to $4\,\mathrm{kHz}$ with an anti-alias filter prior to annotation. For the annotation process, three independent working engineers, familiar with the data, audibly and visually evaluated all of the 191 1D-DUS recordings using a bespoke graphical user interface written in MATLAB (MathWorks, Natick, MA, USA), as described in [27]. This interface subdivided the recordings into segments lasting 0.75 s each. Following this segmentation, annotators categorized each segment into one of six distinct classifications:

• **Interference**: Dominated by electrical disturbances, often manifesting as buzzing noises. These generally originated from other mobile phones.
• **Silent**: Segments that were either barely audible or entirely silent, largely due to the audio cable being only partially inserted into the Doppler device.
• **Talking**: Segments potentially containing audible heartbeats but also featuring human speech or environmental sounds, such as animal noises. This was largely due to the audio cable being only partially inserted into the phone, thereby not triggering the internal/external microphone switch-over.
• **Poor**: Noises that did not align with the characteristics of the other defined categories. These can be due to movement of the mother, fetus or midwife, or poor siting of the Doppler device.
• **Good**: Segments dominated by clear audible heartbeats devoid of the presence of any categories mentioned above.
• **Unsure**: Segments containing mostly reasonable data, but also a mix of other sounds that annotators found challenging to place in a definitive category.

These categories were selected based on their prevalence and utility in guiding midwives to identify issues and make decisions during data collection.

**Figure 3.**
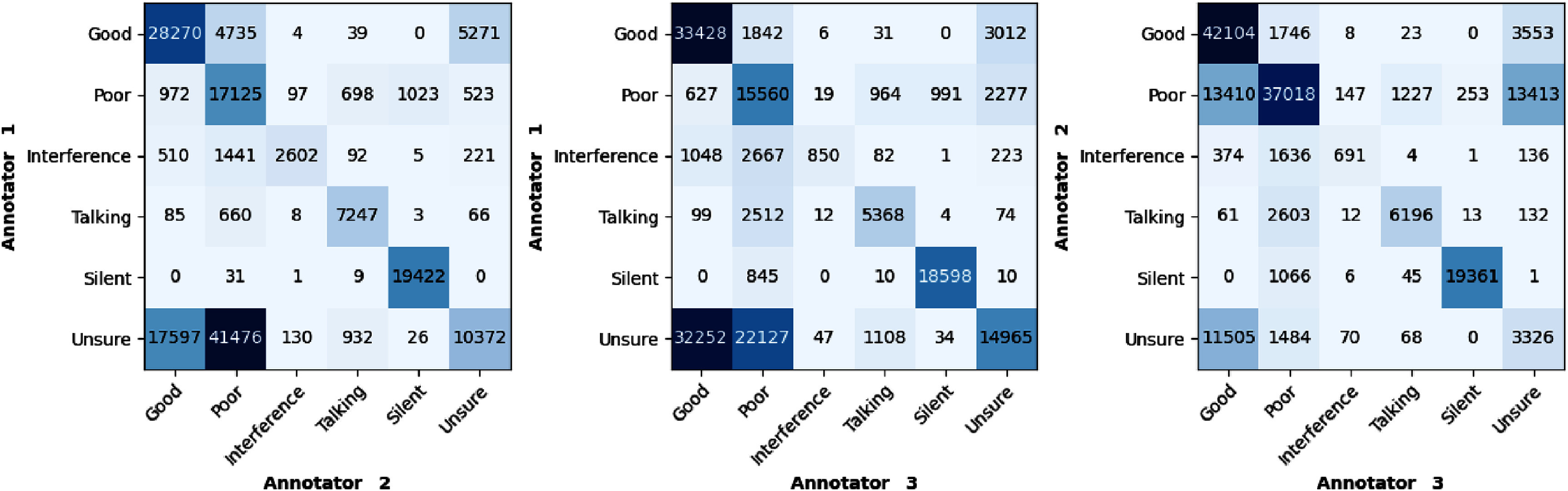
Confusion matrices illustrating agreement among pairs of annotators in the classification of 0.75 s segments across different quality classes in the Guatemala dataset.

The confusion matrices presented in figure 3, illustrate the inter-annotator agreement for the quality categorization of 0.75 s segments into different classes among pairs of annotators. Fleiss’ kappa, which measures overall agreement among the annotators, was found to be 0.45.

#### 2.2.2. Leipzig dataset

Two annotators were responsible for labeling the 5 Leipzig 1D-DUS recordings. The annotation process for this dataset followed the same protocol as for the Guatemala dataset, with the primary difference being the length of the segments used for signal quality labeling: here, each 3.75 s segment was labeled, in contrast to the 0.75 s segments used for Guatemala.

**Figure 4.**
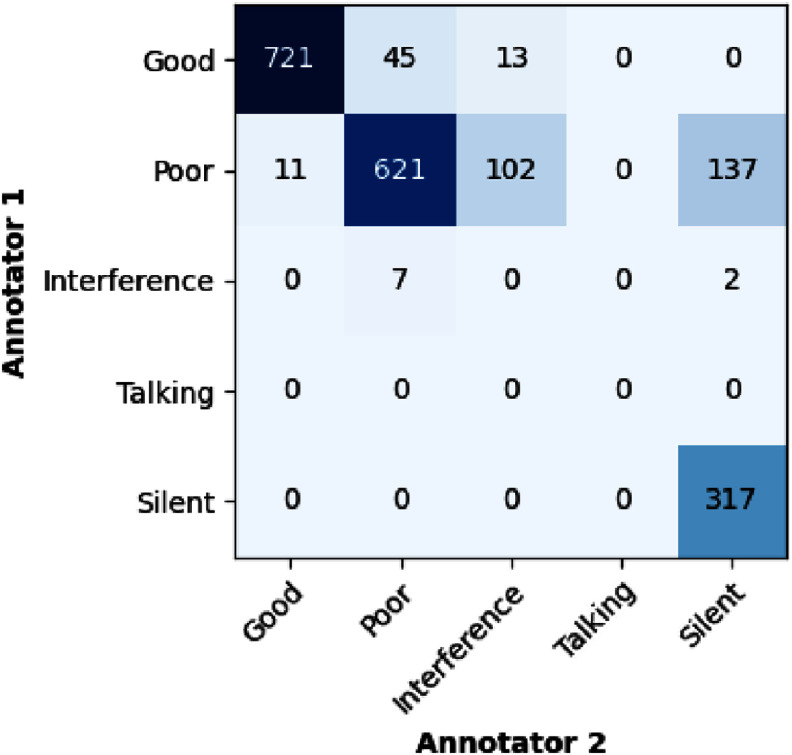
Confusion matrix illustrating agreement among annotators in the classification of 3.75 s segments across different quality classes in the Leipzig dataset.

Figure 4 presents a confusion matrix illustrating the agreement between annotators on segment quality classifications. Cohen’s kappa score was computed to be 0.76. Notably, the categories ‘Poor’ and ‘Silent’ showed the greatest discrepancies between annotators. This variability is understandable, as the distinction between these two categories often relies on subjective perception; some annotators label a window as ‘Silent’ only when no sounds are detected, whereas others may also consider a window ‘Silent’ if they cannot discern a heartbeat amid background noise.

### 2.3. Data preparation

#### 2.3.1. Guatemala dataset

In concordance with the previous studies, signal quality in this research was categorized using 3.75 s windows [26, 27]. (3.75 s is the standard window for heart-rate estimation in CTG, with a good trade-off between sufficient data and stationarity [20].) Each of these windows was composed of five consecutive 0.75 s signal segments (individually annotated). There was a noted variance in the categorization of the $0.75\,\mathrm{s}$ segments among the three annotators. Therefore, segments were only incorporated into the ‘Good’, ‘Poor’, and ‘Silent’ categories if there was unanimous agreement among all three annotators. However, for the ‘Interference’ and ‘Talking’ categories, due to a limited number of segments, those that were labeled similarly by at least two of the annotators were utilized. The total $0.75\,\mathrm{s}$ segments for the ‘Good’, ‘Poor’, ‘Interference’, ‘Talking’, and ‘Silent’ categories were 26 103, 14 796, 2178, 5043, and 18 588, respectively. Any segments that were categorized as ‘Unsure’ were subsequently removed from the analysis.

After identifying the 0.75 s segments agreed upon by the annotators, as described in section 2.2.1, 3.75 s windows were formed by combining five consecutive segments of the same class. To ensure optimal data utilization, a $0.75\,\mathrm{s}$ segment could be repeated in various 3.75 s windows, leading to potential overlaps between these windows. By incorporating this overlap, it was ensured that the maximum information is extracted from the data, especially from the boundaries of each segment, which might otherwise be overlooked or underrepresented. This approach was similar to that used in the previous study [27]. Table 1 presents the distribution of the available 3.75 s windows for each class.

After extracting segments of a 3.75 s duration, a scalogram was generated using the Morlet mother wavelet. This scalogram offers a two-dimensional representation of a signal, illustrating the progression of its frequency components over time. The scalogram was then normalized using its minimum and maximum values. This normalized scalogram served as the input for the DL model, which is discussed in section 2.4.

#### 2.3.2. Leipzig dataset

In the Leipzig dataset, each 3.75 s window was labeled for quality class. Therefore, the data preparation process for this dataset involved extracting the 3.75 s windows that were agreed upon by the two annotators for their quality classification, and creating and normalizing the scalogram for each, similar to the method used for the Guatemala dataset. The number of available 3.75 s windows for each class is presented in table 1.

### 2.4. Network architecture

The deep neural network model implemented in this study was influenced by a previous model employed for estimating gestational age from 1D-DUS signals [21]. We adopted the same framework here, as it has been shown to capture physiologically important characteristics of 1D-DUS from non-physiological ones. Moreover, future iterations of the model may be designed for multi-task functions, specifically using a shared framework to simultaneously identify gestational age, signal quality, and fetal heart rate. Finally, we note that by converting the final model to a TFLite model, we enable the code to easily be ported to an edge device, such as a mobile phone.

The 1D-DUS signals naturally encapsulate the cyclic nature of fetal activity. Components such as wall motion, heart valves, and blood flow exhibit varying velocities and intervals. This results in diverse magnitudes of recorded Doppler frequency shifts, with each component occurring at unique time intervals in relation to others. Given this complexity, scalograms are a natural choice to capture both frequency variations and their temporal relationships, serving as the input for our neural network. The design is structured around three core components, inspired by the beat encoder network explored in earlier research: 1) a feature extractor using a CNN architecture to pinpoint specific frequency-time patterns; 2) an RNN to discern temporal or sequence-based patterns; and 3) an attention mechanism. The attention mechanism predominantly focuses on high-quality segments of the signal,as demonstrated in prior work [21], offering the potential for effective signal quality classification.

**Figure 5.**
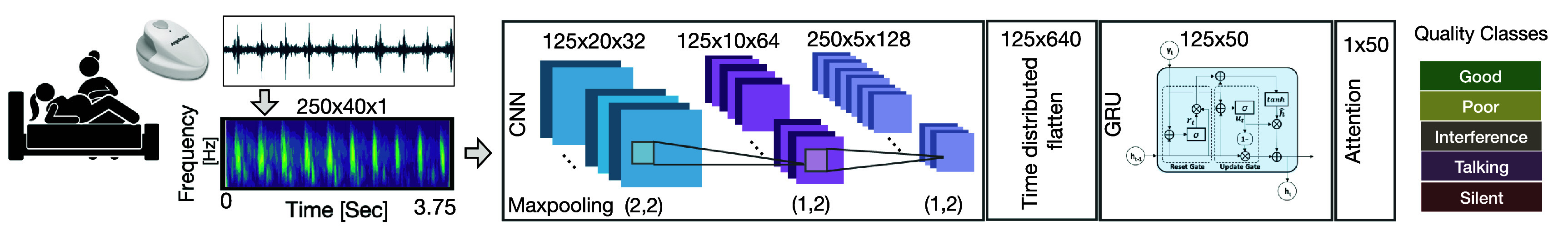
Architecture of the deep learning model for classifying the quality of fetal Doppler signals recorded during home visits by traditional Indigenous midwives using a hand-held Doppler transducer. The scalogram of each 3.75 s of data was calculated and used as an input of the model consisting of CNN and GRU layers, followed by an attention mechanism.

The details of the proposed network are illustrated in figure 5. In this model, scalograms derived from 3.75 s signal windows using the Morlet wavelet with scales between 1 to 40. Each scalogram represented the signal in a two-dimensional format with 250 time scales and 40 frequency bins. The frequency bins ranged from 81.25 Hz to 3250 Hz, capturing a wide spectrum of frequency features. The created scalograms were then processed using a CNN. The initial architecture includes a 2D convolutional layer with 32 $3\times3$ filters, activated by the ReLU function, followed by batch normalization, $2\times2$ max-pooling, and a 25% dropout. This pattern, with the convolutional filters doubling from 32 to 64 and then 128, is repeated in subsequent blocks. In later stages, only the frequency dimension is halved using $1\times2$ max-pooling, preserving the temporal features.

Subsequent to the convolutional blocks, a TimeDistributed layer is used to flatten the output for sequential processing. A gated recurrent unit (GRU) with 50 units then processes these sequences. The GRU uses two gating mechanisms, namely the reset gate *r* and the update gate *z* to control the flow of information. Given the input at time *t*, *x_t_*, the mathematical formulations for these operations in the GRU can be expressed as:

\begin{equation*} \begin{aligned} z_t &amp;= \sigma(W_z x_t + U_z h_{t-1} + b_z), \\ r_t &amp;= \sigma(W_r x_t + U_r h_{t-1} + b_r), \\ \tilde{h}_t &amp;= \tanh(W_h x_t + U_h (r_t \odot h_{t-1}) + b_h), \\ h_t &amp;= (1 - z_t) \odot h_{t-1} + z_t \odot \tilde{h}_t, \end{aligned} \end{equation*}

where *h* represents the hidden state of the GRU, which retains the memory of past inputs and influences future inferences. The symbol *σ* represents the sigmoid activation function, and $\odot$ indicates element-wise multiplication. The parameters *W*, *U*, and *b* are the weight matrices and bias vectors, respectively, that are specific to each operation.

Following the GRU, a dense layer with 50 units, activated by ReLU, processes the data. An attention network is then utilized, offering attention mechanisms to emphasize significant parts of the sequence. The equations governing this attention mechanism are given by:

\begin{equation*} \begin{aligned} u_t &amp;= \tanh(W h_t + b), \\ \alpha_t &amp;= \frac{\exp(u_t^T u)}{\sum_t \exp(u_t^T u)}, \\ v &amp;= \sum_t \alpha_t h_t. \end{aligned} \end{equation*}

In the attention mechanism, the hidden representation at time *t*, denoted as *h_t_*, undergoes a non-linear transformation to produce *u_t_*. This *u_t_* is then compared with a trainable context vector *u*, shared across all time steps, using the dot product to assess the relative importance of each segment in the sequence. From this, the attention weights *α*_*t*_ are derived via a softmax function. Consequently, the vector *v* is computed as a weighted sum of the *h_t_* states based on these attention weights. This vector *v*provides a comprehensive representation, encapsulating the essence of the 1D-DUS input signal.

Finally, the model incorporates a dense layer with 5 units activated by a softmax function, classifying data into the five classes described in section 2.2.

### 2.5. Model evaluation

Based on the network presented in section 2.4, we assessed the performance using three distinct models. In the first model, referred to as CNN+Att, only the CNN network combined with an attention layer was utilized. The second model, GRU+Att, employed solely the GRU layer integrated with the attention mechanism. In the final model, CNN+GRU+Att, we incorporated all components, consisting of the CNN and GRU networks, along with the attention mechanism. This comparative analysis aids in discerning the most influential components of the network and identifying the most streamlined model yielding optimal results.

For the evaluation of each network, we applied a stratified five-fold cross-validation. The stratification was based on the individual recordings, ensuring each recording appeared in only a single fold, thus preventing information leakage of the same recording between training and test sets. (Here we assume noise is independent between recordings, which is a good first-order approximation as they are taken on different days). To guarantee the presence of all labels within each fold, the mode of segment classes for each recording was determined, and stratification was then executed based on these modes.

The networks were developed using TensorFlow 2.11.0 and Python 3.9. The computational system, equipped with 64 GB of RAM, a single CPU, and an NVIDIA Tesla P100 GPU, served for both training and testing the model. The categorical cross-entropy was selected as the loss function, and mini-batch stochastic gradient descent was employed to fine-tune the network parameters. Batch sizes of 128 were utilized. Given the imbalanced distribution of class labels, as presented in table 1, the classification accuracy was enhanced by generating balanced batches through random oversampling, in which minority-class samples were randomly duplicated so that each mini-batch contained an equal number of samples from all classes. This strategy is conceptually similar to applying higher loss weights to minority classes, but was chosen because it guarantees balanced input at each training step, which can improve training stability.

The performance of each model, once trained for each fold, was assessed by the average and standard deviation of precision, recall, and $F1$ score for every quality class over the 5 folds. In addition, the values of micro and macro $F1$ scores were computed. The micro $F1$ score provides a comprehensive measure of a model’s performance across all categories. This is achieved by summing the total number of true positives, false positives, and false negatives from all categories, and then computing the precision, recall, and, subsequently, the $F1$ score from this aggregated data. It is calculated as:

\begin{equation*} \text{Micro F1} = \frac{2 \times \text{Micro Precision} \times \text{Micro Recall}}{\text{Micro Precision} + \text{Micro Recall}}, \end{equation*}

where:

\begin{equation*} \begin{aligned} &amp;\text{Micro Precision} = \frac{\sum_{i=1}^5 TP_i}{\sum_{i=1}^5 TP_i + \sum_{i=1}^5 FP_i} \\ &amp;\text{Micro Recall} = \frac{\sum_{i=1}^5 TP_i}{\sum_{i=1}^5 TP_i + \sum_{i=1}^5 FN_i}. \end{aligned} \end{equation*}

Conversely, the macro $F1$ score computes the $F1$ score separately for each of the *N* = 5 quality categories and then averages them:

\begin{equation*} \text{Macro F1} = \frac{1}{N} \sum_{i=1}^N \text{F1}_i \end{equation*}

In these equations, TP , FP , and FN represent true positives, false positives, and false negatives, respectively.

### 2.6. Comparison with prior work

After identifying the best-performing network structure, its performance was compared with the results of the classifier model from prior research [27]. This comparison was particularly intriguing because that model utilized the same annotated dataset and targeted the same quality classes for classification. It is insightful to determine if the DL model could offer more accurate classifications.

In the previous approach, [27] employed a two-step classification process. Initially, a logistic regression model was trained to identify silent segments based on signal variance. Subsequently, a multiclass SVM was employed to categorize the remaining four classes. For this SVM classification, the best 17 signal-extracted features were utilized. Henceforth, this model is known as ‘the SVM model’ for the purpose of this study.

In [27], the dataset was divided into two equal subsets: one comprising 1D-DUS recordings from 73 subjects for training and the other from 73 subjects for testing. For a rigorous comparison, we aligned our training and testing datasets with those of the SVM model approach. Nonetheless, since 4 of these recordings were unavailable −3 from the training set and 1 from the testing set—our model was trained on data from 70 subjects and tested on recordings from 72 subjects. Consequently, there were 98 recordings for training and 93 for testing. It’s worth noting that a single fetus can be represented in the dataset (at different gestational ages), taken over different visits. While this creates some moderate correlation, it is not significant when considering the noises are generally independent between visits. We also evaluated the SVM model on the same test set and compared the precision, recall, and $F1$ score of each quality category as classified by both models.

### 2.7. Assessing model generalization

To further assess the generalization capability of our model, we tested it on the Leipzig dataset which comprises a total of 5 recordings. Each recording is segmented into non-overlapping windows of 3.75 s. Our model was applied to each window to determine the quality class label. Further details on the performance of the model are provided in section 3.3.

### 2.8. Mobile edge-based implementation for real-time classification

We implemented a DL algorithm and developed an Android application using Kotlin to enable real-time classification of DUS signals. The application was deployed on a Google Pixel 6a smartphone running Android version 14.0. It integrates Python-based functions for data preprocessing and utilizes TFLite for executing the DL model.

To evaluate the performance of the developed application, we conducted three sets of experiments. Initially, we assessed the performance of the Kotlin code on the Google Pixel 6a. For this evaluation, we randomly selected 250 DUS signal segments, each lasting 3.75 s, from the Guatemala dataset (described in section 2.1.1). These segments were categorized using the Python version of the code on a MacBook Pro (Apple M1 silicon CPU, 16GB RAM, 512GB SSD hard drive, 2022) laptop into 110 ‘Good’, 78 ‘Poor’, 11 ‘Interference’, 17 ‘Talking’, and 34 ‘Silent’. The segments were subsequently transferred and stored on the internal storage of the Google Pixel 6a. The application directly accessed these segments and performed quality classification, with the results then compared to those obtained from the MacBook Pro.

In the second experiment, we investigated the impact of hardware designed to link the Doppler device to the smartphone on signal quality. For this, the same 250 segments were replayed as audio files via a MacBook Pro, with the audio output connected to the Pixel 6a using a CableCreation adapter (3.5 mm audio jack to USB-C). This setup mimicked the actual hardware connection intended for use with the Doppler device, except that in practice, the audio would be transmitted from the Doppler device rather than the laptop. The application on the mobile device processed each segment individually, classifying the quality of each streamed segment. These classifications were then compared with the outputs from the Python code on the laptop.

In the third experiment, we evaluated the real-time performance of the application. A ring buffer was implemented within the app to manage incoming data streams effectively. We randomly selected five 3.75 s audio segments from each quality category (25 total), concatenating them ten times to create a continuous 37.5 s signal for each category. Similar to the second experiment, these audio signals were played via the MacBook Pro, and the audio output was again connected to the Pixel 6a using the CableCreation adapter. The application assessed the quality of the 3.75 s chunks in real-time, classifying each segment every second after the initial 3.75 s, resulting in 34 evaluations per signal. The model’s output probability for each quality class was computed for each segment and averaged to determine the most probable quality class. These results were then compared with the quality classifications obtained from the laptop.

## 3. Results

### 3.1. Model assessment

Table 2 displays the average $F1$ scores alongside their standard deviations obtained over a 5-fold cross-validation for the five quality classes across three distinct network architectures: CNN+Att, GRU+Att, and CNN+GRU+Att. From the results, the comprehensive CNN+GRU+Att model had higher average $F1$ scores in the ‘Good’, ‘Poor’, and ‘Silent’ quality classes compared to the other two models. However, the CNN+Att model—comprising CNN and attention mechanisms—achieved superior $F1$ scores in the ‘Interference’ and ‘Talking’ classes. A potential reason for this could be the limited data available for these classes (as presented in table 1), causing the more complex model to overfit the training data and thus negatively impacting accuracy on the test set. Among the models, the GRU+Att (integrating RNN and attention) showed the lowest performance across all categories.

**Table 2.** Mean $F1$ scores (in %) of the three studied models across five categories, assessed via five-fold cross-validation, along with micro and macro averages of the $F1$ scores (standard deviations provided in parentheses). Bold shows the highest score for each category across models.

	CNN+Att	GRU+Att	CNN+GRU+Att
Good	98.21 (0.72)	97.25 (1.08)	**99.16 (0.30)**
Poor	89.12 (9.27)	86.23 (7.89)	**94.11 (3.55)**
Interference	**87.54 (4.86)**	65.81 (13.35)	84.22 (6.63)
Talking	**96.79 (0.98)**	84.69 (6.15)	94.88(4.76)
Silent	94.44 (7.54)	97.39 (3.02)	**98.79 (1.81)**
Micro $F1$	95.12 (4.00)	93.47 (2.78)	**97.41** (1.24)
Macro $F1$	93.22 (3.14)	86.27 (3.40)	**94.23** (2.44)

For the subsequent analyses, the best model was identified by comparing the average micro and macro $F1$ scores obtained from the five-fold cross-validation, as illustrated in table 2. The CNN+GRU+Att model exhibited the highest performance with average micro and macro $F1$ scores of 97.41% and 94.23%, respectively, indicating its superior classification efficacy. Consequently, the CNN+GRU+Att model was selected as the best model and was employed for further evaluation.

For a more in-depth insight into the CNN+GRU+Att model’s classification capability across various quality categories, table 3 provides precision, recall, and $F1$ score values, averaged over the 5-fold cross-validation. To complement this, a cumulative confusion matrix that aggregates classifications across the five folds is provided in table 4. The ‘Good’ class emerged as the most accurately classified category, with all metrics exceeding 99%. In contrast, the ‘Interference’ was the most challenging class, with an $F1$ score of 84%, which is possibly attributed to its minimal training labels as highlighted in table 2. The confusion matrix further illustrates that the erroneously classified ‘Interference’ segments predominantly fall under the ‘Good’, ‘Poor’, and ‘Talking’ categories.

**Table 3.** Precision, recall, and $F1$ score values for the CNN+GRU+Att model, averaged over five-fold cross-validation. Numbers in parentheses indicate standard deviation. Bold values indicate the highest performance across categories for each metric.

Category	Precision (%)	Recall (%)	$F1$ score (%)
Good	$ \textbf{99.17} (0.69)$	$99.15 (0.37)$	$ \textbf{99.16} (0.30)$
Poor	$96.41 (0.77)$	$92.22 (6.95)$	$94.11 (3.55)$
Interference	$88.28 (12.84)$	$83.21 (11.65)$	$84.22 (6.63)$
Talking	$93.98 (8.36)$	$96.23 (2.23)$	$94.88 (4.76)$
Silent	$97.83 (3.32)$	$ \textbf{99.83} (0.19)$	$98.79 (1.81)$

**Table 4.** Cumulative confusion matrix over five-fold cross-validation for the CNN+GRU+Att model.

	Estimated class					
Actual class		Good	Poor	Interference	Talking	Silent

	Good	21 831	109	56	12	0
	Poor	72	9106	66	297	333
	Interference	85	89	1396	50	6
	Talking	29	113	32	5790	11
Silent	0	24	0	3	17 233

**Figure 6.**
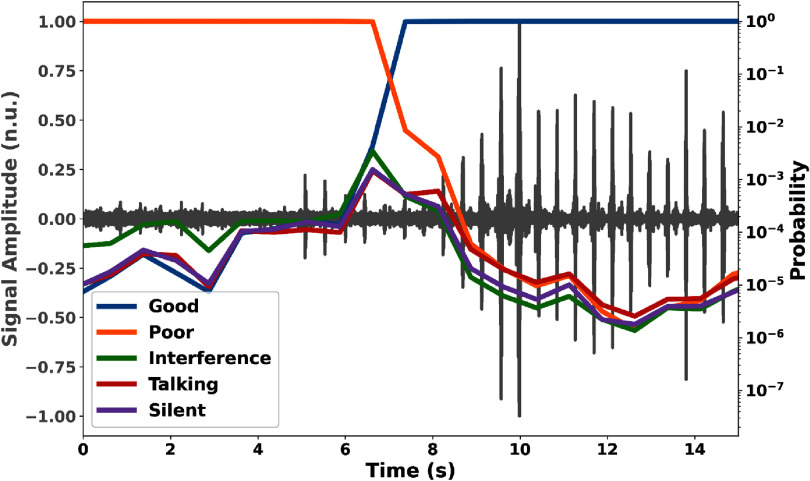
Example of a 15 s Doppler ultrasound signal segment, plotted in gray, and the corresponding quality class probabilities estimated over time by the CNN+GRU+Att model. The left *y*-axis displays the normalized signal amplitude, and the right *y*-axis, set to a logarithmic scale, shows the class probabilities.

In addition, figure 6 illustrates 15 s of Doppler signals from the validation set, along with the probability of each class over time, as estimated by the CNN+GRU+Att model. For this analysis, windows of 3.75 s were extracted with a stride of 0.75 s, and the probability of each signal quality class was estimated for each window. In the first approximately 6 s, the signal was of poor quality, evidenced by low heartbeat amplitudes relative to the noise level, and the model estimated the highest probability for the ‘Poor’ quality class. Subsequently, as the signal exhibited robust heartbeats with higher amplitudes, indicating improved signal quality, the probability of the ‘Good’ quality class increased, demonstrating the model’s effective performance in quality classification.

### 3.2. Evaluating DL and SVM on common data

The selected CNN+GRU+Att network, hereafter also referred to as the DL model, was trained on the same dataset as the SVM model presented by [27], as detailed in section 2.7. Table 5 compares the precision, recall, and $F1$ score results of both the DL and SVM models evaluated using an identical test set.

**Table 5.** Comparison of precision, recall, and $F1$ scores for the deep learning (DL) model from the current study and the support vector machine (SVM) from [27] across five Categories in the test set. Bold values indicate the highest performance across categories for each metric.

*Category	Precision (%)		Recall (%)		$F1$ score (%)	
	DL	SVM	DL	SVM	DL	SVM
Good	**99.36**	97.06	**98.79**	93.97	**99.07**	95.49
Poor	**95.21**	86.05	**94.15**	91.42	**94.68**	88.65
Interference	64.40	**85.13**	81.44	**92.44**	71.93	**88.63**
Talking	**98.17**	95.87	93.69	**96.63**	95.88	**96.25**
Silent	96.84	**100.00**	**99.98**	99.91	98.39	**99.96**

The DL model surpassed the SVM model in all metrics for the ‘Good’ and ‘Poor’ classes. Notably, the classification performance for the ‘Good’ category was especially very high, with an $F1$ score exceeding 99%. However, the SVM model exhibited better capability in categorizing signals with ‘Interference’. The performance metrics for ‘Talking’ and ‘Silent’ categories were close for both models, but the SVM marginally outperformed the DL model in terms of $F1$ score.

### 3.3. Testing on an additional dataset

The trained DL model was tested on the Leipzig dataset, and the different metrics across all five categories are presented in table 6. Given the absence of ‘Talking’ and ‘Interference’ categories in this dataset, the metrics cannot be computed for these two classes and have been denoted by N/A in table 6. Notably, the model achieved a high $F1$ score of 93.28% for the ‘Good’ category. In addition, it demonstrated the highest accuracy and recall in the ‘Poor’ quality class, along with the highest precision in the ‘Silent’ category. The confusion matrix for this dataset is outlined in table 7.

**Table 6.** Performance of the deep learning model on the Leipzig dataset across five categories. Bold values indicate the highest performance across categories for each metric.

Category	Accuracy (%)	Precision (%)	Recall (%)	$F1$-score (%)
Good	90.43	96.31	90.43	**93.28**
Poor	**91.47**	76.24	**91.47**	83.16
Interference	N/A	N/A	N/A	N/A
Talking	N/A	N/A	N/A	N/A
Silent	62.46	**100.0**	62.46	76.89

**Table 7.** Confusion matrix for the deep learning (DL) model on the Leipzig dataset.

	Estimated class					
Actual class		Good	Poor	Interference	Talking	Silent

	Good	652	66	0	3	0
	Poor	25	568	0	28	0
	Interference	0	0	0	0	0
	Talking	0	0	0	0	0
Silent	0	111	4	4	198

### 3.4. Real-time classification performance

The quality classification of the 250 DUS segments on the Pixel 6a, conducted in the first experiment, exactly matched the output from the Python version of the code running on a Macbook, confirming the accuracy of the Android app implementation. The mean execution time for the classifier on each 3.75 s segment running on the Pixel 6a was 233.9 ms, with a standard deviation of 5.4 ms, and a maximum execution time of 277.0 ms. In the second experiment, (transmitting audio from the Macbook Pro M1 to the Pixel 6a) a single (‘Poor’ quality) segment was misclassified (as ‘Interference’). This misclassification likely resulted from the filtering characteristics of the adapter, which introduced distortion into the audio signal. Moreover, this difference is unimportant, since the segment would be rejected either way. Additionally, in the third experiment, the real-time analysis using a ring buffer on the 25 recordings created by concatenating ten 3.75 s segments showed 100% consistency with the actual quality labels of the segments, validating the robustness and accuracy of the mobile application for real-time signal quality assessment.

## 4. Discussion

### 4.1. Performance and limitations of the CNN+GRU+Att model

The CNN+GRU+Att model demonstrated its robustness in comparison to the simpler architectures, such as CNN+Att and GRU+Att. One notable strength of the CNN+GRU+Att model is its ability to capture both local features, via the CNN layers, and temporal dependencies through the GRU layers. This simultaneous feature extraction likely contributed to its superior performance in the ‘Good’, ‘Poor’, and ‘Silent’ classes. Conversely, the GRU+Att model had the lowest performance across all classes. This highlights that solely focusing on temporal relationships may be inadequate. The inclusion of CNN layers appears essential for identifying patterns within the signals.

Nevertheless, the CNN+GRU+Att model struggled to outperform the simpler CNN+Att model in the ‘Interference’ and ‘Talking’ classes. This implies that model complexity, while beneficial, can pose risks, especially when training data is scarce, leading to potential overfitting. It is worth noting that the labeled data for the ‘Interference’ and ‘Talking’ categories might also be noisier (with lower inter-rater agreement levels) than the other three. As described in section 2.2, the annotations for the ‘Good’, ‘Poor’, and ‘Silent’ classes were used under conditions where all three annotators were in agreement. Yet, due to data scarcity for ‘Interference’ and ‘Talking’, labels were considered even with the consensus of only two annotators. This observation emphasizes the vital importance of having robust and sufficient training data for DL models to achieve effective generalization. Additionally, as discussed in section 2.2 and illustrated in figure 3, ‘Interference’ was the most challenging label for annotators, showing the least agreement among them. This discrepancy is also reflected in the model’s weakest performance in classifying this category.

The model’s decent classification performance on the Leipzig dataset, captured separately from the training data, further emphasizes its capability in quality classification. This external validation is crucial, demonstrating the model’s generalizability across different data sources. In this evaluation, the classification for the ‘Good’ quality class was notably high. However, as depicted in table 7, some labels from the ‘Silent’ class were misclassified as ‘Poor’. This is not entirely unexpected, given the lower agreement between annotators for these classes, as illustrated in figure 4. The ‘Silent’ and ‘Poor’ classes were often annotated interchangeably, which contributed to diminished model accuracy in these categories.

### 4.2. DL vs SVM in quality classification

The comparison between the DL model and the SVM model of [27], highlights the advantages of the DL approach. Notably, the DL model achieved an $F1$ score of 99.07% for the ‘Good’ class, outperforming the SVM model (at 95.45%), as detailed in table 5. It is essential to accurately detect these high-quality DUS signal segments, as they are the foundation for further analyses and are vital for effective fetal health monitoring. While recognizing unwanted signal categories, such as ‘Interference’, ‘Talking’, and ‘Silent’, is informative for the traditional Indigenous midwives during signal recording, the primary focus remains on differentiating between good and low-quality signals. Adjustments in hardware configurations can mitigate issues like ‘Interference’, ‘Talking’, and ‘Silent’. However, low-quality data resulting from suboptimal Doppler usage presents a more challenging obstacle. This challenge underscores the primary objective of this study: offering AI-assisted training to navigate these issues. This further emphasizes the advantage of the DL model for signal quality classification.

Additionally, it is important to note that, although the evaluation metrics associated with the DL model for the classification of ‘Silent’ DUS segments appear slightly inferior to the SVM approach, the DL model might still excel under general conditions. The SVM model employs a simple logistic regression based on signal variance to identify silent signals. In this method, any signal with variance below a set threshold is labeled as ‘Silent’. However, this threshold may not be consistent when the DUS signal is obtained using different devices or in varied application settings. The DL model, with its capacity to identify patterns and relationships in the raw signal, offers a more adaptable and universally relevant classification mechanism. This flexibility enhances its potential for a wide variety of recording environments.

### 4.3. Implications and future directions

The successful implementation and real-time testing of our DL algorithm in an Android app for DUS signal quality classification mark a significant advancement in point-of-care decision support tools. The consistency in classification results across different testing environments underscores the reliability of our approach. Although the adapter introduced a misclassification in one segment, the system demonstrated consistently high performance, underscoring its robustness. We acknowledge that the filtering characteristics of the adapter and their variability across different cables, adapters, and devices may influence signal properties and model performance. Future studies with larger datasets should therefore systematically evaluate the impact of hardware-related filtering. Moreover, the confusion between interference and noise is not important in the context of down-stream processing.

The maximum execution time of 277 ms per segment is much less than the user-required update interval of one second. Real-time processing on mobile devices is therefore possible (with room for several other algorithms), which is crucial for providing immediate feedback to midwives and other healthcare providers in low-resource settings, enabling them to make informed decisions during data collection. At the same time, we acknowledge that the clinical value of this signal quality feedback has not yet been demonstrated. Although the algorithm is currently being tested in the field with promising preliminary results, the next step will require prospective field evaluations to establish whether real-time quality assessment translates into sustained improvements in data quality and, ultimately, contributes to better maternal and neonatal outcomes. As such, the present study should be viewed as a technical advance that supports, but does not yet confirm, broader clinical impact. Future research may also assess the utility of this approach during labor, where maintaining consistent and high-quality Doppler recordings is essential for accurate fetal monitoring and timely clinical decision-making.

Furthermore, due to the scalability of DL models, enhanced performance is anticipated as more annotated data is collected, particularly for the ‘Interference’ class with the lowest accuracy. In this study, a clear correlation was observed between the quantity of available data in each class and the model’s performance for that class. The model performed better on classes with a larger number of labels. For example, the performance metrics with the highest values, as presented in table 3, were exhibited by the ‘Good’ and ‘Silent’ classes, which had the greatest number of labeled data (as referenced in table 1). Additionally, when comparing the outcomes of a model trained on a smaller dataset, there was a more pronounced performance dip for classes with fewer examples. A comparison between tables 5 and 3 reveals that the $F1$ score for ‘Interference’ dropped from 84.22% to 71.93%. Consequently, enhanced model performance is expected by gathering more data, especially for classes with fewer examples, such as ‘Interference’ and ‘Talking’. Future research will focus on training the model using a broader collection of recordings.

Moreover, the designed architecture is compatible with other DL-based algorithms, such as those for estimating gestational age and blood pressure. Our approach therefore provides a general platform for the creation of a multi-task algorithm, which will be the focus of our future work.

Finally, the proposed model, for real-time 1D-DUS signal quality assessment introduces two iterative learning schemes. The first involves incremental retraining of the model in the light of new data. The user can label data in real-time, primarily through objecting to the classifier’s output. This label is then saved with the raw data for later relearning. This approach also ensures the model’s adaptability across diverse data collection settings or environments. The other form of retraining is from the machine learning model to the user. Over time, the user will become more adept at perceiving the audible qualities of good quality data, and become better at ‘locking on’ to an improved signal.

Supplementary video demonstrates the mHealth kit and its usage in the field by Indigenous midwives in rural Guatemala. This video showcases the system components described in this article and its operation under real-world conditions.

## 5. Conclusions

In this study, we presented a DL model for the real-time classification of 1D Doppler ultrasound signal quality, tailored for low-resource settings and integrated into an mHealth application. These recordings were captured by traditional Indigenous midwives with minimal training via an affordable Doppler device connected to a smartphone. Specifically, the model was found capable of classifying $3.75\,\mathrm{s}$ segments of the signals into five categories with high accuracy. The accuracy of this model surpassed that of conventional machine learning algorithms previously used, particularly in the classification of ‘Good’ quality signals, achieving an $F1$ score of over 99%. Additionally, the model’s effectiveness was validated by evaluating its performance on 1D-DUS recordings captured independently from the training data, emphasizing its robustness and generalizability. Our experimentation involved deploying the algorithm on a smartphone, yielding rapid and consistent classification results, showcasing its potential for real-time applications. This algorithm provides a reliable tool for integration into smartphone apps used by midwives and other front-line providers, enabling real-time feedback on the quality of recorded data. This ensures that better data will be available for future analysis, aiding in the improvement of fetal health in resource-poor settings.

Notably, the system has been integrated into an mHealth platform used by midwives in Guatemala [18, 19]. Future work will focus on evaluating the impact that the AI-driven tools have in terms of improved data quality and improved outcomes. Moreover, there exists potential for further refinement of the models presented in this article through the acquisition and annotation of additional DUS signals, potentially by the midwives at the point of acquisition. While this study establishes the technical feasibility of real-time Doppler signal quality assessment, demonstrating its clinical impact will require prospective validation studies assessing outcomes in real-world maternal care settings.

## Data Availability

It is scheduled for release alongside an upcoming public competition. As part of the grant aims, we will make the dataset publicly available through the annual PhysioNet Challenge, which is led by Dr Clifford. The data that support the findings of this study are available upon reasonable request from the authors.
